# Comparative Study of Essential Oils Extracted from Algerian *Myrtus communis* L. Leaves Using Microwaves and Hydrodistillation

**DOI:** 10.3390/ijms13044673

**Published:** 2012-04-12

**Authors:** Baya Berka-Zougali, Mohamed-Amine Ferhat, Aicha Hassani, Farid Chemat, Karim S. Allaf

**Affiliations:** 1 Group of Intensification of Transfer Phenomena on Eco-Processes for Industry, Laboratory of Engineering Science for Environment/FRE 3474 CNRS, Pole Science and Technology, University of La Rochelle, Avenue Michel Crepeau, 17042 La Rochelle cedex 01, France; 2 Laboratory of Research on Bio-active Products and Valorization of Biomasse, Ecole Normale Supérieure, vieux-Kouba 16050, Alger, Algeria; 3 Group of Research on Eco-Extraction of Natural products GREEN, Laboratory of Security and Quality of Vegetal Products/UMR A 408 INRA, Université d’Avignon et des Pays de Vaucluse UAPV, F-84029 Avignon, France; 4 ABCAR-DIC Process, 40, rue Chef de Baie 17000 La Rochelle, France

**Keywords:** *Myrtus communis* L., essential oils, solvent free microwave extraction SFME, hydrodistillation, antioxidant activity, antimicrobial activity

## Abstract

Two different extraction methods were used for a comparative study of Algerian Myrtle leaf essential oils: solvent-free-microwave-extraction (SFME) and conventional hydrodistillation (HD). Essential oils analyzed by GC and GC-MS presented 51 components constituting 97.71 and 97.39% of the total oils, respectively. Solvent-Free-Microwave-Extract Essential oils SFME-EO were richer in oxygenated compounds. Their major compounds were 1,8-cineole, followed by α-pinene as against α-pinene, followed by 1,8-cineole for HD. Their antimicrobial activity was investigated on 12 microorganisms. The antioxidant activities were studied with the 2,2-diphenyl-1-picrylhydrazyl (DPPH^•^) radical scavenging method. Generally, both essential oils showed high antimicrobial and weak antioxidant activities. Microstructure analyses were also undertaken on the solid residue of myrtle leaves by Scanning Electronic Microscopy (SEM); it showed that the SFME-cellular structure undergoes significant modifications compared to the conventional HD residual solid. Comparison between hydrodistillation and SFME presented numerous distinctions. Several advantages with SFME were observed: faster kinetics and higher efficiency with similar yields: 0.32% dry basis, in 30 min as against 180 min for HD.

## 1. Introduction

Common Myrtle belongs to the Myrtaceae family with some 145 genus and over 5500 species [1]. It is mainly spread around the Mediterranean, including the Middle East and many other countries in Southern Europe. It is also found in Asia, New Zealand, America, Southern Russia and Australia [2,3]. This shrub with leathery hermaphrodite, persistent and with aromatic leaves has a longevity of over 300 years. Generally, it is found at an altitude not exceeding 800 m. When in flower, it emits a characteristic aroma of mixed incense and honey. It is often associated with forests of oak, mastic and Aleppo pine. It grows equally well on limestone or silica, and easily adapts to many soils. It is fairly resistant to cold and readily acclimatizes to hot weather.

In Algeria, the wild plant known as “Al-Rihan” or “el-halmouche” grows very well in many areas, on mounds or hills, in coastal or in more remote areas. The Myrtle plant is currently generating real interest regarding its use as a medicinal plant in Algeria. Myrtle flowers, leaves and berries are used for external applications to heal wounds, for skin diseases (psoriasis, herpes, bruises *etc*.) and for internal functions to treat many diseases such as dysentery, urinary tract infections, hemorrhoids, and even hair loss. In some areas, its use is recommended to lower blood sugar as well as to improve digestion. However its main use is for the treatment of respiratory problems [4,5].

Algerians often use fresh or dried myrtle berries while estimating their nutritional properties despite their astringency and taste Moreover, in Corsica [2] and Sardinia [6], they are used to make a popular digestive liqueur recognized as having interesting stomachic qualities and also as a substitute for hops for making beer [2]. In the past, they were used as black pepper substitute [3] and to decorate some dishes [7]. Other varieties of myrtle berries are white, less astringent and slightly sweet. In addition, the myrtle has many biological properties (hypoglycemic, antimicrobial, analgesic, antioxidant *etc*. Furthermore, as far as we know, no acute toxicity has been reported, excepting a study published in 1979 [8], which noted some toxicity in rats; the therapeutic range for humans is considered safe [9].

A considerable number of studies have been devoted to the extraction of myrtle essential oils EO, and there has been an increasing interest whatever the part of the plant (leaves, seeds, berries, stems and flowers) [2,9–11]. These numerous investigations have suggested that the chemical composition of myrtle essential oil greatly depends not only on the zones [12], seasons [13], and the plant organs [11,13–15] but also on the extraction method [16,17]. Whatever the traditional method used in industry (hydrodistillation, steam extraction, solvent extraction), essential oil extraction is a high-energy consumption unit operation and hence an environmental unfriendly process. Reducing energy consumption and ensuring sustainable development are pushing researchers and engineers into finding innovative solutions to improve essential oil extraction methods, and at the same time being eager to enhance its quality and to better control its composition.

On the one hand, in standard extraction processes such as steam extraction or hydrodistillation, energy consumption is correlated with the energy needed to evaporate volatile compounds, the heating of the material, and importantly the amount of added water. HD is a high-temperature EO extraction process requiring a very long treatment time (up to 24 h), with a great amount of added water (about six liters water/kg of raw material [18]. Such a situation requires high energy consumption and the prolonged contact between the plant and water at high temperatures may cause changes in the EO chemical composition as well as generating hydrolysis [19]. It thus results in very high amounts of contaminated residual water and unrecovered final solid waste.

On the other hand, extraction using organic solvents seems to be a very simple way to extract essential oils. However, the solvent extraction may induce both loss of the most volatile compounds and extraction of some non-volatile compounds, which may result in modification in effectiveness and change in quality of EOs. Of necessity it involves a second stage of distillation, which greatly increases energy consumption. The principal weakness of this operation lies in the requirement of completely eliminating the solvent from the extract [20].

Moreover, typical organic solvents may be very toxic to human health and generate many safety problems as they are often volatile and flammable. Their complete elimination is often faced with quite stringent and difficult standard regulations. All these technical and environmental problems have led to the necessity to define new free solvent extraction methods able to minimize the pollution risk. As conventional methods can hardly meet all the requirements, non-organic solvents such as water or ionic liquids as well as supercritical CO_2_ have been proposed. Although the latter is normally very appropriate in terms of quality and environment, its high cost often makes it inaccessible or limited to high value products.

As hydrodistillation and steam extraction are simultaneous heat and mass, external and internal, successive transfer processes, the operation kinetics must depend strictly on the lowest, limiting process. Therefore, to decrease energy consumption and even more so to control quality, various intensification processes have been investigated. The internal heat and mass transfer depends largely on the intrinsic material proprieties that operators cannot modify. Only a grinding process can permit a reduction in size and processing time while maintaining the same diffusivity and thermal conductivity [21]. However, the long processing time of essential oil steam extraction is in the main part due to a paradoxical situation [22]. In this situation, heat and mass transfers having as a driving force the temperature gradient and the partial pressure gradient of EOs, respectively, are both directed from the surface to the core of the product.

To completely modify this situation, some researchers [22] proposed instant autovaporization through instant controlled pressure drop (DIC). Other authors planned using microwaves as a means of heating. Instant autovaporization by dropping the pressure towards a vacuum allows EO mass transfer with the gradient of total pressure as driving force (Darcy-type law); thus EOs flow from the core of the material with high total pressure of vapor to its surface under vacuum [23]. It has been proven that this innovative method of instant autovaporization can dramatically reduce extraction time, much improving quality while reducing energy consumption, and preserving the environment. The recovered condensates are usually very stable oil-in water emulsions with droplet diameters less than 0.5 μm [24].

Moreover, as far as we know, no work has been undertaken using a solvent free microwave-extraction (SFME) method to extract EOs from myrtle. However, numerous industrial operations have more and more favored using microwave energy for heating wet materials, with as specificity the unique capability to penetrate deeply into materials, thus allowing a substantial reduction in processing time, often by as much as one to ten times. Out of this unique property of deep energy penetration many other advantages arise. Microwave (MW) heating compared to various conventional heating methods seems usually to be a more economical process. Indeed, the use of microwaves assures a reduction in the heat energy lost towards the surrounding medium. As a deeply heating process, MW enables the surface to be kept at a lower temperature than the level needed by pure contact/conduction or by Infrared IR, which can save more energy than convection. The major advantages of microwave heating can be summarized as a higher heating rate, increased safety, improved ecology, and better product quality.

A large number of publications concerning MW confirms the exceptional value of this new technique and shows its great potential for the extraction of natural compounds. Its application areas are extensive and cover a wide range of products ranging from organic synthesis to combinatorial chemistry [25]. In heating-extraction of volatile molecules such as essential oils, from microwave treatment, benefits such as improved quality and increased yields have been expected. All research works in this field, have exhibited acceleration of kinetics and improvement of quality; the chemical composition of the essential oils was similar to that of the fresh plant without any process contamination [26].

## 2. Results and Discussion

### 2.1. Kinetics of Hydrodistillation and Microwave Extraction

**Figure 1:**
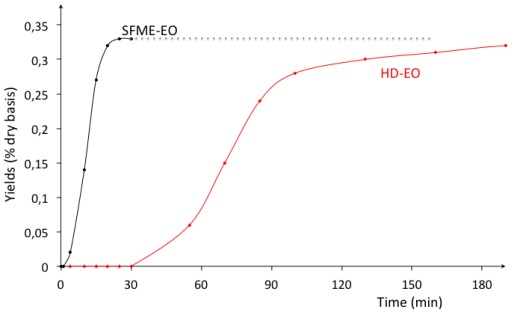
Kinetics of essential oil extracted by hydrodistillation (HD-EO) and Solvent-Free-Microwave-Extraction (SFME-EO) from fresh leaves of myrtle.

Figure 1 represents the HD and SFME extraction kinetics of the cumulative EO compounds extracted from the fresh myrtle leaves.

These results show a SFME extraction rate dramatically higher than HD’s, with a complete extraction achieved in less than 30 min for SFME extraction as against 180 min in the case of HD. The model already explained in Equation 4 could predict such results:a long processing time was needed in the case of HD because of the progressive front kinetics due to the paradoxical coupled mass and heat transfers; while a main part of mass transfer with the SFME essential oil extraction is performed with the gradient of total pressure as driving force. However, no significant difference in yields could be signaled between SFME-EO and HD-EO. One can note that Microwave heating seems to imply negligible rupture of secretion of the organ walls.

### 2.2. Physical Characteristics of Essential Oils

Physical constants (relative density, refractive index and optical rotation) of the myrtle EO obtained by HD and SFME are listed in Table 1.

The optical rotation *θ* was determined for a concentration of 10 mg/mL of HD-EO and SFME-EO. Note that for myrtle EO, there is no official standard. Accordingly, it was not possible to compare on that basis. Mean values are given for information. According to the values in Table 1, it is of note that only the optical rotation shows a significant difference for the two types of EO, while the relative densities and the refractive indices are similar for both oils.

### 2.3. Analysis of HD-EO and SFME-EO

#### 2.3.1. Analyses of HE Obtained by Hydrodistillation

The average yield obtained on the basis of three successive extractions was 0.32 ± 0.06 g EO/100 g dry basis. The essential oil obtained was yellow, very light, and almost colorless with a mobile aspect. It smelled fresh and pleasant.

Besides traces, 45 compounds were identified in HD-EO; they represented 97.39% of the total essential oils. They were dominated by hydrocarbon monoterpenes (51.36%), which were represented mainly by α-pinene (44.62%) followed by limonene (3.71%). The oxygenated monoterpenes were present at 37.47%, with 1,8-cineole as their major component (25.46%). Of note is also the presence of 4-terpineol, α-terpineol and linalool with 1.33%, 2.16%, and 2.07%, respectively as well as other oxygenated compounds (6.03%) such as acetates, phenols and more particularly geranyl acetate (2.23%) and methyl eugenol (2.22%) with other oxygenated monoterpenes (1%). The sesquiterpenes, hydrocarbon (4.74%) and oxygenated (2.86%), were less represented than the monoterpenes, with compounds each below 1%.

#### 2.3.2. Solvent-Free-Microwave-Extraction SFME

The microwave essential oils SFME-EO were almost colorless, with a clear appearance. Their fresh and very pleasant fragrance evoked that of eucalyptus. The average yield obtained from three trials was 0.33 ± 0.08 g EO/100 g db; similar to HD-EO.

SFME-EO showed 49 compounds of more than 0.10% (Table 2); they represented 97.71% of the overall composition of the essential oil. Compared to HD-EO, hydrocarbon monoterpenes significantly dropped (36.30% as against 51.36%), especially α-pinene (30.65 as against 44.62%), hydrocarbon and oxygenated sesquiterpenes equally decreased (3.04% as against 4.78% and 1.89% as against 2.86%, respectively). In contrast, the oxygenated compounds, especially the monoterpenes, clearly increased (55.23% as against 37.47%); with 1,8-cineole (32.12% as against 25.46%), 4-terpineol (2.89% as against 1.33%), the α-terpineol (3.01%), the α-terpinyl acetate (1.15%), geranyl acetate of (3.17%) but particularly methyl eugenol (6.02% as against 2.22%). Thus, it is to note that the chemical composition of essential oils is very dependent on the extraction method.

**Figure 2:**
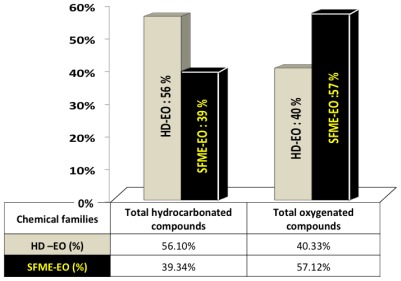
Total oxygenated compounds and non-oxygenated compounds obtained by hydrodistillation (HD-EO) and Solvent-Free-Microwave-Extraction (SFME-EO) from fresh leaves of myrtle.

Besides, the microwave extraction of the volatile compounds from green Myrtle leaves showed an inversion of the major compound, which became 1.8-cineole instead of α-pinène. As shown in Figure 2, the SFME extraction increased the total of oxygenated compounds (40.33 to 57.12%) associated with a reduction in hydrocarbon compounds from 56.10% to 39.34%.

As presented in Table 3, the composition of essential oils strictly depends on the origins and the extraction processes. In the Algerian myrtle leaf essential oils, whatever the extraction method used, α-pinene and 1,8-cineole are the major compounds, corroborating that with Tunisian myrtle [11,12] as well as French myrtle [12]. For Spain [27] and Greece [7] myrtle, the EO major compounds are myrtenyl acetate and 1,8-cineole. However, the three major compounds from Morocco, Yugoslavia and Lebanon fresh myrtle leaf essential oils are α-pinene, 1,8-cineole and limonene [12]. Shikhiev *et al*. [28] reported that the essential oil of Azerbaijan myrtle can be distinguished by four major compounds that are limonene, linalool, the α-pinene, and 1,8-cineole.

It was also reported [10], that four major compounds characterize the myrtle essential oils of Iran: α-pinene, limonene, 1.8-cineole, and linalool. These results corroborate those obtained by Yadegarinia *et al*. [29]. Ghasemi *et al*. [17] conducted another comparative study for HD and SFE (supercritical CO_2_) extraction carried out on Iranian myrtle. Seven major compounds were identified in HD-EO: α-pinene, 1,8-cineole, limonene, β-linalool, α-terpinolene, linalyl acetate, and α-terpinyl acetate; the yield was 0.47% (*v/w*). The extract obtained by SFE CO_2_ with the addition of methanol as an organic modifier contained the major compounds α-pinene, 1,8-cineole, limonene, β-linalool and linalyl acetate; the yield of the extract was 6.3% (*w/w*).

The major compounds of essential oil extracted from Albanian leaves of myrtle [15], were 1,8-cineole, α-pinene and limonene, as well as myrtenyl acetate and linalool. In the case of Turkish myrtle [3], three major compounds were found: 1,8-cineole, linalool, and myrtenyl acetate.

Concerning myrtle from Portugal, Pereira *et al*. [30] cited three major compounds whose dominance is closely linked to growth cycle. These were myrtenyl acetate, α-pinene, and limonene with 1,8-cineole.

Recently, in the case of Egyptian myrtle Nassar *et al*. [9] reported that the major compounds of the EO were 1,8-cineole (27.19%) immediately followed by α-pinene (25.53%). The composition of the HE is enriched by the presence of β-linalool (11.75%), *p*-menth-1-enol (6.95%), myrtenyl acetate (4.2%), linalyl acetate (3.39%), neryl acetate (2.94%), limonene (1.6%) and α terpinyl-acetate (1.4%).

Algerian myrtle was, recently, investigated using instant controlled pressure drop (DIC) technology [4] to directly extract the essential oil from the leaves of myrtle; this extraction process achieved in a very short time (2 min) led to a yield similar to that obtained by steam distillation; but the extract was particularly enriched with oxygenated monoterpenes (52.83%) mainly containing 1,8-cineole (21.76%). Hydrocarbon monoterpenes were present at 26.00% with α-pinene as the major compound, (23.33%).

### 2.4. Impact of Extraction HD and SFME on the Cell Structure

Scanning electron microscopy (SEM) micrographs were performed on the surfaces and cross sections of raw material and residual HD and SFME treated leaves. It was noted that the differences in structural changes observed with HD *vs*. SFME were due to the different extracting modes used.

Indeed, while untreated leaves have regularly arranged and tidy cells, cross-sections of SFME residual leaves show large cavities and great structural changes on secreting cells emptied of essential oil.

The HD residual leaf views show modifications only located on the surface with wrinkled appearance similar to that from an intense spin. HD treatment did not result in major changes to the cross section secreting and normal cell structure.

### 2.5. Results of the Antioxidant Activities

The antioxidant capacities of the essential oils and the antioxidants taken as reference were evaluated by the minimum concentration IC_50_ required to inhibit 50% of DPPH free radicals. The rate of reduction or inhibition of the initial DPPH^•^ I% was calculated as follows:

(1) $$\text{I}\;(\%)=\frac{\left({\text{A}}_{\text{control}}{-\text{A}}_{\text{sample}}\right)}{{\text{A}}_{\text{control}}}\%$$

Where Acontrol was the initial DPPH^•^ absorbance without extract, and Asample was the initial DPPH^•^ absorbance with the presence of the test compound (EO). Extract concentration providing 50% inhibition (IC50) was calculated from the linear regression algorithm of the graph, which was plotted as inhibition percentage against concentration of EO or references. The values were expressed in micrograms of extract per millilitre of methanol solution. All measurements were performed in triplicate and grouped together in Table 4.

Both extracts showed antioxidant activity, however, significantly lower than those of the reference antioxidant gallic acid and quercetin. The fresh myrtle leaf essential oil obtained by SFME had a slightly higher antioxidant activity than that obtained by HD. It is also of note that the values found show some discrepancies with those in the literature [7,11]. The reduction of DPPH radical depends on the nature, structure and concentration of the antioxidant compounds [32–36]. It may also be affected by light, oxygen and pH of the reaction [37]. To have a consistent and meaningful comparison, the experimental conditions must be absolutely identical.

It is not easy to establish the specific effect of each of the numerous essential oil compounds and the possible synergy of such very complex mixtures. It seems possible that a small concentration of a specific compound could be essential in determining the overall antioxidant and biological activities. However, one can note that SFME-EO has better antioxidant activities and a greater proportion of oxygenated compounds (57.12% as against 40.33% for HD-EO) and higher phenolic compounds (6.34% as against 2.39% for HD-EO). This conclusion corroborates with Jukic’ & Milos [30] finding that the antioxidant activities of essential oils are as high as the phenol compound content.

### 2.6. Results of Antimicrobial Activity

The values of minimum inhibitory concentration (MIC) of essential oils were determined by using 12 microorganisms: three gram-positive and five gram-negative (four multi-resistant bacteria), one yeast and three filamentous fungi. The strains used in this study were pathogenic or toxigenic to humans or to plants. MIC values obtained for the two essential oils are shown in Table 5.

All extracts showed antimicrobial activity regarding both gram positive and gram-negative individual bacteria. Note that significant inhibitory activity toward the highly resistive bacteria such as *Klebsiella pneumoniae* (E52), *Salmonella enterica* (E32) and *Enterobacter Cloaca* (E13), was observed with the two essential oils; however, it was more pronounced in the SFME-EO probably because of the higher content of oxygenated and phenolic compounds [38]. The effectiveness of both essential oils was the same for the yeast *Candida albicans* (API 200). Despite some resistance when compared to other strains, the MIC is still quite significant, as it does not exceed 50 μL/mL. The fungi *Aspergillus ochraceus* and *Fusarium culmorum* showed greater sensitivity for SFME-EO.

Despite the difference in behavior of the two essential oils, it was not possible to precisely recognize the compounds that should have the greatest antimicrobial activity. Several authors did not attribute it to specific molecules thus attributing antimicrobial activity to a set of molecules that work synergistically. However, it is proven that antimicrobial activity of essential oils is closely related to their chemical composition [39].

Whatever the extraction both HE and SFME methods, the essential oils of fresh leaves of Algerian myrtle could inhibit the growth of bacteria, fungi and yeasts. Thus, they present a broad spectrum of antimicrobial activity. Let us finally note that many studies have focused on the fact that essential oils had more impact on gram-positive bacteria than gram-negative ones [40]. This resistance is due to the fact that gram-negative bacteria have a wall associated with an outer complex membrane although poor in peptidoglycan. This slows down the passage of EO hydrophobic compounds [41].

## 3. Fundamental

### 3.1. Paradox of Coupled Heat and Vapor Transfers

Allaf *et al*. [42] studied the paradox of coupled heat and mass transfer processes during a standard steam distillation. Mass transfer normally depends on the nature and localization of liquid and vapor within the plant as a porous medium. It may be conducted by diffusion reinforced by capillary force for liquid/solid interaction, and by a similar DARCY process within the holes for gas/solid interaction. In steam extraction, Allaf *et al*. [23] assumed that the mass transfer is a gas phase diffusion, which can be shown as a Fick-type law related to the partial pressure gradient of each volatile compound through an effective diffusivity *D*_eff_. Thus, the kinetics depends on several factors such as the compound nature and thermodynamic properties, the temperature, structure and morphological state of the plant (degree of porosity, specific surface area, permeability of secretion element walls *etc*.). Nonetheless, the main involvement of the structure is due to the permeability of secretion of the element walls, acting as the main barriers. In similar cases, essential oil extraction needs an amount of heat flowing within the porous material to change the liquid into gas phase [22]:

(2) $$\dot{Q}-\vec{\nabla }\cdot \vec{\phi }=\rho_{s}(c_{ps}+Ec_{ps}+Wc_{pw})\frac{\partial T}{\partial t}+\alpha \frac{\partial \left(p_{e}\frac{M_{e}L_{e}}{RT}+p_{w}\frac{M_{w}L_{w}}{RT}\right)}{\partial t}$$

where *α* stands for “absolute expansion rate”, *E* for essential oil content dry basis, *W* for water content dry basis, ϕ⃗ for the heat flow, *c*_ps_, *c*_pe_, and *c*_pw_ for heat capacities of solid, essential oils and water, respectively, *M*_e_ and *M*_w_ for molar mass of essential oils and water, respectively, *L*e and *L*w for evaporation latent heats of essential oils and water, respectively, and, finally, *p*_e_ and *p*_w_ for the partial pressures of vapor of essential oils and water, respectively. Whatever the method adopted with the exception of microwave heating, internal heat transfer is achieved by conduction:

(3) $$\vec{\nabla }\cdot (\lambda \vec{\nabla }T)=\rho_{s}(c_{ps}+Ec_{ps}+Wc_{pw})\frac{\partial T}{\partial t}+\frac{\partial }{\partial t}\left[\frac{\alpha }{RT}(p_{e}M_{e}L_{e}+p_{w}M_{w}L_{w})\right]$$

As external heating is obtained by saturated steam convection and condensation, Allaf *et al*. [23] assumed that the repartition of temperature quickly reached a stationary state. Internal heat transfer was then exclusively used to evaporate essential oils and water:

(4) $$\vec{\nabla }\cdot (\lambda \vec{\nabla }T)=\frac{\partial }{\partial t}\left[\frac{\alpha }{RT}(p_{e}M_{e}L_{e}+p_{w}M_{w}L_{w})\right]$$

Fick’s law types can control the internal transfers of essential oil and water vapor. They are separately shown through Allaf’s formulation [43]:

(5) $$\frac{p_{e}/T}{\rho_{s}}({\vec{v}}_{eo}-{\vec{v}}_{s})=-D_{{eff}_{e}}\vec{\nabla }\left(\frac{p_{e}/T}{\rho_{s}}\right)\;\;\;\text{and }\;\;\frac{p_{v}/T}{\rho_{s}}({\vec{v}}_{v}-{\vec{v}}_{s})=-D_{{eff}_{v}}\vec{\nabla }\left(\frac{p_{v}/T}{\rho_{s}}\right)$$

By assuming that *ρ_s_* = *constant*and V⃗*_s_* = 0, Equation (5) becomes:

(6) $$\left(p_{e}/T\right){\vec{v}}_{eo}=-D_{{eff}_{e}}\vec{\nabla }\left(p_{e}/T\right)\;\;\;\text{and }\;\;\left(p_{v}/T\right){\vec{v}}_{v}=-D_{{eff}_{v}}\vec{\nabla }\left(p_{v}/T\right)$$

As the external steam is saturated, one can approximately assume the internal vapor partial pressure *p*_w_ within the porous material is constant. Equation (4) becomes:

(7) $$\lambda \vec{\nabla }\cdot (\vec{\nabla }T)=\alpha M_{e}L_{e}\frac{\partial }{\partial t}\left(\frac{p_{e}}{RT}\right)$$

With *x*-coordinates, homogeneous and isotropic particles allow the Equations (6,7) to be transformed into:

(8) $$\left(p_{e}/T\right)v_{eo}=-D_{{eff}_{e}}\frac{\partial \left(p_{e}/T\right)}{\partial x}\;\;\;\text{and }\;\;\lambda \frac{\partial^{2}T}{\partial x^{2}}=\alpha M_{e}L_{e}\frac{\partial }{\partial t}\left(\frac{p_{e}}{RT}\right)$$

On the other hand, it is well known that usually (*p_e_/T*) is higher as the temperature is raised:

$$\frac{\partial \left(p_{e}/T\right)}{\partial T}>0$$

As the exchange surface defined by *x* = 0 has the highest temperature value, heat flow should be directed along the positive *x*-axis *x* > 0:

(9) $$\frac{\partial T}{\partial x}<0,\frac{\partial \left(p_{e}/T\right)}{\partial x}<0\Rightarrow v_{eo}>0$$

The result is a paradoxical situation implying an essential oil flow completely opposite to the mass transfer required for the required extraction operation. In this case, the usual essential oil steam extraction is achieved by “progressive front” kinetics.

One can adopt other ways such as the DIC process for heating and moving volatile molecules; the extraction operation is then much more intensified. Another possible way appears to be the microwave essential oil extraction.

### 3.2. Thermal Effect of Electromagnetic Field

Allaf *et al*. [42] studied the Microwave heating mechanism as a coupled heat generation based primarily on the dipole behavior of the polar molecules in interaction with microwaves, and the heat conduction within the material. In the absence of electric fields, polar molecules are randomly oriented. They rapidly change their orientations in reaction to the changing electric fields. Heat is generated as a result of the rotation of the molecules. Microwave heating closely depends on the physical state and electromagnetic properties of the material, which are characterized by the complex relative permittivity ɛ^*^ and the complex relative permeability μ^*^, where:

(10) $${ɛ}^{*}={ɛ}^{\prime }-i{ɛ}^{\prime \prime }\;and\;\mu^{*}=\mu^{\prime }-i\mu^{\prime \prime }$$

The real permittivity, or dielectric constant *ɛ′*, characterizes the penetration of microwaves into the material while ɛ″, which is the loss factor, indicates the material’s ability to store the electrical potential energy, and the loss tangent tan δ designates the ability of the material to convert absorbed energy into heat. *ɛ′* significantly depends on microwave frequency and material temperature. For optimum coupling, a balanced combination of moderate ɛ′, to permit adequate penetration, and high loss (maximum ɛ″ and tan δ) is required. In a microwave essential oil extraction operation, heat generation and transfer within a plant as a porous medium is described by the following Equation:

(11) $$\dot{Q}-\vec{\nabla }\cdot \vec{\phi }=\rho_{s}(c_{ps}+Ec_{ps}+Wc_{pw})\frac{\partial T}{\partial t}+\alpha \frac{\partial \left(\frac{M_{e}L_{e}p_{e}+M_{w}L_{w}p_{w}}{RT}\right)}{\partial t}$$

where the second term in LHS stands for heat transfer, which normally is performed through the conduction law. The first term in RHS stands for the sensitive heat accumulation and the second term for the heat dissipated for evaporation.

The first term in LHS *Q*&*dot;* stands for the microwave volumique absorbed power or, in other words, the heat produced per unit volume per unit time; it is also called absorption rate density (ARD) and can be expressed by the following relation:

(12) $$\dot{Q}(\vec{r})=\pi f{ɛ}_{o}{ɛ}^{\prime \prime }(\vec{r})|E(\vec{r}){|}^{2}$$

where |*E*(*r*)| is the amplitude of electric field intensity, which varies depending on the point defined by the vector (*r⃗*). If the thickness *l*_p_ of the heated material is sufficiently large and the loss factor is constant, power distribution can follow Lambert’s law:

(13) $$\dot{Q}(x)=\pi f{ɛ}_{o}{ɛ}^{\prime \prime }|E_{o}{|}^{2}exp\left(-\frac{x}{d_{p}}\right)$$

where |*E*_0_| stands for the amplitude of electric field intensity at the material surface (*x* = 0), and *d*_p_ for the penetration depth [29]:

(14) $$d_{p}=\frac{c}{2\pi f\sqrt{2{ɛ}^{\prime \prime }}}{\left(\sqrt{1+{\left(\frac{{ɛ}^{\prime \prime }}{{ɛ}^{\prime }}\right)}^{2}}-1\right)}^{-1/2}$$

At the penetration depth *d*_p_ from the surface (*x* = 0), the power density is equal to 36.788% of the power density on the surface.

As microwave heating is distributed *versus* the position within the wet porous material through Equation 13, the temperature hardly reaches a stationary level and can easily exceed the high value that assures the internal vapor pressure is higher than the external surrounding pressure. During this stage, a main part of mass transfer of both water and essential oil vapors is performed through a Darcy law type with the total pressure gradient as driving force. Usually, essential oil compounds have partial pressures much lower than water; at atmospheric pressure, their boiling point is known to be more than 220 °C. The presence of water inside the plant during microwave extraction of volatile molecules is necessary because water molecules are normally the unique polar molecules and also because the contribution of water to the total internal pressure is indispensable for assuring a Darcy’s type transfer. Thus, the paradoxical stage is absent; only in the part of material where *Q*(*x*) is assumed to be negligible, may it exist. Thanks to its muchy higher extraction rate and because of the absence of hydrolysis degradation and as the high temperature is reached in a short time, SFME presents numerous advantages compared with hydrodistillation, in terms of both process performance such as faster kinetics, higher efficiency, lower costs, lower environmental impact, and better extract quality.

## 4. Materials and Methods

### 4.1. Reagents and Chemicals

2,2-Diphenyl-1-picrylhydrazyl (DPPH 98%); tert-butylated hydroxytoluene (BHT ≥ 99%); 3,4,5-trihydroxybenzoic acid (gallic acid ≥ 99%); quercetin (≥98%) and anhydrous sodium sulphate (≥99%) were provided by Sigma-Aldrich (St. Quentin Fallavier, France). Analytical grade methanol and hexane were provided by Carlo Erba (Val de Reuil, France).

### 4.2. Reagents and Chemicals

Raw material samples were collected during the full fruiting period of myrtle. The aerial parts were harvested early in the morning, in mid-November 2007 in the forest of Bainem, located in the suburbs northwest of Algiers. This site is about 400 m above sea level; its location is very sunny and far from any pollution, with a mild climate and good weather. It has dense vegetation with varied multi-woody and herbaceous species.

The plant was identified by the head of the herbarium of the National Institute of Agronomy (INA) of Algiers.

### 4.3. Processing Protocol

**Figure 3:**
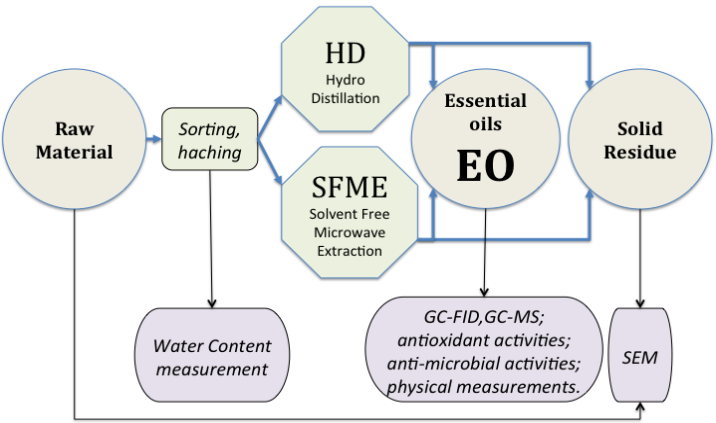
Processing and assessment protocol.

Figure 3 presents the various stages of the treatment.

#### 4.3.1. Determination of the Initial Moisture

In this study, only the leaves were used. Their moisture content was determined from three separate samples of 3 g of fresh leaves of myrtle. They were coarsely chopped and placed in small cups with a thin layer to be dried in an infrared balance model Mettler Toledo LP-16 (Bishop International Akron, OH, USA). The moisture content was 48.40 ± 0.20 g H_2_O/100 g fresh material or 93.80 ± 0.75 g H_2_O/100 g dry matter.

#### 4.3.2. Hydrodistillation

A quantity of 200 g of fresh leaves of myrtle was coarsely chopped and immersed in 1.5 L of distilled water contained in a 3 L flask. Distillation was performed using a modified Clevenger’s glass apparatus. The extraction process was carried out for 3 h after the first drop of distillate until complete exhaustion of the plant. The distillation started after a heating time of 40 min. The condensation was carried out continuously with water chilled to 5°C. Trials were performed with three successive repetitions. The essential oils extracted were recovered, dried with anhydrous sodium sulphate, stored in a refrigerator at 4 °C in tightly closed amber vials, away from contamination sources and collected prior to use for analysis and various functional biological tests.

#### 4.3.3. Solvent Free Microwave Extraction (SFME)

**Figure 4:**
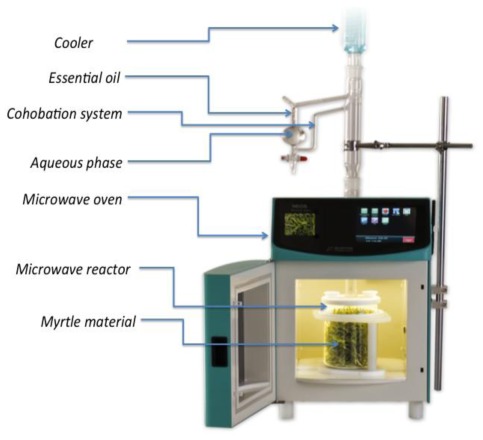
Microwave—Clevenger.

200 g of fresh leaves of myrtle, coarsely chopped, were introduced into a 500 mL glass reactor, already described by Ferhat *et al*. [26] (Figure 4). It is an improved 2450 MHz multimode microwave modified Clevenger apparatus for distillation of essential oils. The condensation was accomplished through a column continuously cooled with water chilled to 5 °C. Trials were achieved without addition of external water; however continuous recycling of the aqueous phase (cohobation) maintained the plant at its own constant humidity. SFME was performed at 1 kW under atmospheric pressure for a period of 30 min. This time limit was set following a kinetic study previously performed on the raw material. The heating time was 150 s.

Experiments were performed with three successive repetitions of the trials. Each extract was dried over anhydrous sodium sulfate and stored in an amber vial placed in a refrigerator at 4 °C prior to GC analysis.

### 4.4. Experimental Assessments

#### 4.4.1. Scanning Electron Microscope

For a better understanding of the extraction phenomenon and its correlation with inherent structural changes in essential oil secreting cells, the surface structure and control areas of residual solid after HD and SFME essential oil extraction were examined and compared with the fresh myrtle leaves using scanning electron microscope (SEM). These tests were performed at the common center of analysis (CCA) of the University of La Rochelle with a JEOL 5410LV FEI QUANTA 200F model (Philips, Croissy-sur-Seine, France). Structural analyses were carried out under partial vacuum (7PA) and accelerating voltage of 20 kV.

#### 4.4.2. Characterization of Physical and Functional Properties of Myrtle Leaf Essential Oils Extracted by HD and SFME

In addition to the organoleptic characteristics, measurements of physical constants (density, optical rotation and refractive index) were performed at 20 °C in accordance with AFNOR standards. The thermometer was graduated at intervals of 0.5 °C.

The refractive index was measured with reference to the wavelength of the D-line of sodium (*λ* = 589.3 nm).

In this study, tests of antioxidant and antimicrobial activities were undertaken on the essential oil extracts obtained by HD and SFME from fresh myrtle leaves.

##### 4.4.2.1. Measurement of Antioxidant Activity

The antioxidant activities of essential oils extracted from fresh myrtle leaves by HD and SFME were evaluated following the model described by Brand-Williams *et al*. [44] with some changes. The absorbance measurements of various samples were performed using a Helios Omega spectrophotometer model (Thermo Fisher Scientific, St. Herblain, France). Maximum absorption of the radical DPPH was identified at 516 nm in methanol.

A solution of DPPH (0.2 mM) in methanol was prepared as well as a series of EO solutions in methanol from 20 to 5000 μg/mL from a stock solution of 10 mg/mL. The concentrations of BHT, quercetin and gallic acid used as references were located in the ranges between 2 μg/mL and 25 μg/mL, 1 μg/mL and 12 μg/mL, and 0.3 μg/mL and 5.0 μg/mL, respectively. They were made from stock solutions of 50 μg/mL, 30 μg/mL, and 10 μg/mL, for BHT, quercetin and gallic acid, respectively.

A sample of 1.5 mL of DPPH was added to 2.5 mL of diluted essential oil extract. The mixture was vigorously stirred and left for 30 min at room temperature in a dark area, away from light sources. Measurements were carried out with a blank prepared by mixing 2.5 mL of the same essential oil extract with 1.5 mL of methanol. Different antioxidants were used as references following the same procedure for evaluating the antioxidant capacity. Tests were performed with three successive repetitions.

##### 4.4.2.2. Measurement of Antimicrobial Activity

The microdilution method was used to determine minimum inhibitory concentrations (MIC). The antimicrobial activity of EO extracts obtained by HD and SFME was tested against panel of 12 pathological microorganisms which included Gram+ and Gram– bacteria such as *Bacillus subtilis; Staphylococcus aureus; Listeria monocytogenes; Salmonella anterica* (E32); *Klebsiella pneumoniae* (E40); *Escherichia coli* (E52); *Pseudomonas aeruginosa; Enterobacter cloacae* (E13); as well as fungi (*Aspergillus flavus*, *Fusarium culmorum*, *Aspergillus ochraceus*) and yeast (*Candida albicans*).

The microdilution was performed following the method of Oki *et al*. [45] with some modifications. The microorganism concentration was 3 × 10^6^ colony-forming units CFU/mL. The seeding was done in 3 mL sterile Petri dishes.

Each solution including the reference was introduced into a sterile test tube. Then 2.7 mL of nutrient agar was added as the means of growth of the microorganisms. Using a vortex, each tube was vigorously stirred in order to perfectly disperse the EO in the culture medium. The content of each test tube was immediately and consistently poured into a sterile Petri dish for each dilution. The same procedures were used for the reference.

The dishes were then incubated in an oven at 37 °C for 24 h for bacteria and 30 °C for 48 h for fungi and yeast. Each test was performed with three successive repetitions.

#### 4.4.3. Analysis by GC and GC-MS

HD and SFME fresh myrtle leaf essential oils were analyzed by gas chromatography (GC) and also coupled with mass spectrometry GC-MS. The gas chromatograph used was a Varian model 3800 (Cie Varian, Les Ulis; France), equipped with a flame ionization detector and fused silica capillary columns. Analyses were performed with two-column brand Varian, of different stationary phases; one polar (polyethylene glycol) CP WAX 52CB model and the other nonpolar (polydimethylsiloxane) Factor FourVF-5ms model. The two columns were of the same sizes, which were 30 m long, 0.25 mm internal diameter and 0.25 μm stationary phase film thickness.

Prior to analysis all essential oils were diluted ten times with hexane. The analytical conditions of operation were: the carrier gas had a constant helium flow (1 mL/min); the injection and detection temperatures were 250 and 280 °C, respectively; the injected volume was 0.50 μL with a split ratio adjusted to 20:1. The programming of the oven temperature was 50 °C for 8 min, then following a gradient of 2 °C/min until 250 °C; this final temperature was kept constant for 5 min.

A series of *n*-alkanes ranging from C_5_ to C_28_ was injected under the same analytical conditions as the samples.

GC-MS analyses were achieved with a brand Varian, model 3900 (Cie Varian, Les Ulis; France) coupled with a mass spectrometer, model Saturn 2100T, using the same columns and the same analytical conditions as those of the GC-FID.

Fragmentation was performed by 70 eV electron impact. The temperatures of injection, detection, and transfer line were respectively 250, 280 and 250 °C.

The scan set was run over 35–400 amu mass (m/z). The data acquisition was done with a periodicity of 1.2 scan/s. Identification of compounds was performed by comparing some of their retention index (Kovats indices determined from the retention time in the series of *n*-alkanes.) with those of references and the other compounds through their mass spectra from the literature [46–49] or from the database and spectral libraries (Varian 1998 and Saturn NISTMS Database libraries).

Some EO compounds that had very similar retention indices were identified with standards injected under the same conditions.

The percentages of compounds contained in the essential oils were calculated from the chromatograms obtained by GC-FID with the apolar column.

## 5. Conclusion

In this study, it was shown that the chemical composition of the essential oil of Algerian wild myrtle leaves depends on the method of extraction and has also differences compared to those reported in the literature, however with less impact in terms of yields. Many other studies show extreme variability due to geographical location and growth stage.

Solvent Free Microwave extraction SFME was found to be highly effective enabling a considerable reduction in extraction time (30 min against 180 min), providing an essential oil with a chemical composition enriched in oxygenated compounds but with yields similar to those of steam distillations.

SFME-EO retained the note “fresh” similar to the original plant. Moreover, SFME required only the initial water content of the raw material. Thus, it consumed much less energy than hydrodistillation, in both stages of extraction and condensation. The raw material is fully exhausted leading to a semi-dry solid residue, which is easier to evaluate than the waterlogged hydrodistillation residue. These benefits show that SFME is a good alternative to other extraction techniques and microwave technology can thus be recommended for the extraction of natural volatiles.

Both essential oils HD-EO and SFME-EO had a low antioxidant activity. However, the SFME-EO showed a greater antioxidant activity in the DPPH^•^ test. This can be explained by the presence of a greater number of phenolic compounds and oxygenated molecules.

SFME-EO also showed a better antimicrobial activity than HD-EO vis-à-vis all examined microorganisms with, as exception, *Candida albicans* (IPA 200), which showed the same resistance. This performance corroborates Bekhechi’s work and can be attributed firstly to a greater proportion of oxygenates and also to the presence of eugenol and methyl eugenol.

## Figures and Tables

**Table 1:** Physical characteristics of fresh myrtle leaf essential oils extracted by hydrodistillation (HD-EO) and Solvent-Free-Microwave-Extraction (SFME-EO).

Physical characteristics	HD-EO	SFME-EO
Relative density at 20 °C (*d*)	0.9051 ± 0.0342	0.9047 ± 0.0536
Refractive index at 20 °C (*α*)	1.470 ± 0.021	1.471 ± 0.012
Optical rotation at 20 °C (*θ*)	1.54 ± 0.13°	1.86 ± 0.09°

**Table 2:** Chemical composition of essential oils extracted by hydrodistillation (HD-EO) and Solvent-Free-Microwave-Extraction (SFME-EO) from Algerian fresh myrtle leaves with initial moisture of 93.8 g H_2_O/100 g dry matter.

N°	Compounds a	RI b	RI c	HD-EO (%)	SFME-EO (%)
**Monoterpene hydrocarbons**				**51.36**	**36.30**
1	α-Thujene	930	1,030	0.10	0.12
2	α-Pinene	939	1,028	44.62	30.65
3	Sabinene	978	1,128	tr d	0.10
**Monoterpene hydrocarbons**				**51.36**	**36.30**
4	β-Pinene	979	1,116	0.45	0.60
5	β-Myrcene	994	1,162	0.22	0.18
6	α-Phellandrene	1,004	1,175	0.22	0.37
7	δ 3-Carene	1,011	1,146	0.51	0.58
8	α-Terpinene	1,020	1,178	0.16	0.15
9	*p*-Cymene	1,024	1,281	0.25	0.22
10	Limonene	1,029	1,208	3.71	2.22
11	(*Z*)-β-Ocimene	1,043	1,236	0.40	0.40
12	γ-terpinene	1,059	1,263	0.30	0.30
13	α-Terpinolene	1,088	1,290	0.42	0.41
**Oxygenated monoterpenes**				**37.47**	**55.23**
14	1.8-cineole	1,032	1,216	25.46	32.12
15	β-Linalool	1,098	1,552	2.07	2.90
17	trans-PinoCarveol	1,139	1,665	tr d	0.24
18	Borneol	1,168	1,717	tr d	0.51
19	Terpinen–4–ol	1,177	1,610	1.33	2.89
20	α-Terpineol	1,192	1,711	2.16	3.01
21	Geraniol	1,256	1,849	0.42	0.70
22	Linalyl acetate	1,257	1,556	0.37	0.85
23	Methyl citronellate	1,261	1,570	0.11	0.68
24	Exo-2-hydroxycineole acetate	1,343	1,765	0.21	0.17
25	α-Terpinyl acetate	1,352	1,707	0.72	1.61
26	Eugenol	1,360	2,164	tr d	0.21
27	Neryl acetate	1,361	1,723	tr d	0.11
28	Geranyl acetate	1,380	1,761	2.23	3.10
29	Methyl eugenol	1,397	2,030	2.22	6.02
30	(*E*)-Methyl isoeugenol	1,492	2,196	0.17	0.11

**Sesquiterpene hydrocarbons**				**4.74**	**3.04**
31	β-Elemene	1,390	1,600	0.21	0.29
32	β-Caryophyllene	1,419	1,612	0.40	0.33
33	γ-Elemene	1,436	1,637	0.10	0.10
34	α-Humulene	1,454	1,676	0.34	0.32
35	β-Chamigrene	1,477	1,741	0.25	0.19
36	β-Selinene	1,490	1,727	0.88	0.27
37	α-Selinene	1,498	1,729	0.84	0.47
38	δ-Cadinene	1,523	1,763	0.37	0.10
39	Cadina-1.4-diene	1,534	1,789	0.20	0.11
40	Selina-3.7 (11) -diene	1,542	1,786	0.37	0.31
41	Germacrene B	1,558	1,846	0.78	0.55
**Oxygenated sesquiterpenes**				**2.86**	**1.89**
42	Spathulenol	1,578	2,146	0.10	0.12
43	Caryophyllene oxide	1,583	2,008	0.26	0.27
44	Globulol	1,582	2,098	0.88	0.91
45	Cubenol	1,646	2,074	0.18	tr d
46	β-Eudesmol	1,650	2,246	0.24	0.15
47	α-Bisabobol	1,685	2,228	0.28	tr d
48	Juniper camphor	1,698	2,275	0.92	0.44
**Other compounds**				**0.96**	**1.25**
49	(*Z*)-3-Hexenol	856	1,389	0.64	0.67
50	Isobutyl isobutyrate	896	1,086	0.32	0.21
51	Isoamyl 2-Methyl butyrate	1,099	1,296	tr d	0.37
Total hydrocarbonated compounds				56.10	39.34
Total Oxygenated compounds				40.33	57.12
Other compounds				0.96	1.25
Total identified compounds				97.39	97.71

a Essential oil compounds classified by chemical families and percentages were calculated by GC–FID on non-polar VF-5ms capillary column;

b Retention indices calculated through *n*-alkanes (C5–C28) on non-polar VF- 5ms capillary column;

c Retention indices calculated through *n*-alkanes (C5–C28) on polar CP wax 52CB capillary column;

d Traces < 0.10%.

**Table 3:** Percentage composition of main compounds in myrtle leaf oils from various origins.

	Italy	Greece	Croatia	Spain	Algeria		Marocco	Egypt	Iran	Tunisia	Corsica	Azerbaijan	Yougoslavia	Lebano*n*	Iran		Albania	Turkish	Portugal
	[6]	[7]	[13]	[14]	[4]		[12]	[9]	[10]	[12]	[12]	[30]	[12]	[12]	[17]		[15]	[3]	[31]
	
Compounds (%)	HD	HD	HD	HD	HD	DIC	HD	SD	HD	HD	HD	SD	HD	HD	HD	CO_2_	HD	HD	HD

α-pinene	28.9–41.6	10.1–11.6	6.6–16.4	8.9	50.8	23.3	18.5–25.0	25.5	29.4	51.1–52.9	53.5–56.7	14.5	23.8–24.8	32.1	31.8	38.6	19.4–20.3	6.4–9.0	10.4–21.5

p-cymene	0–1.8									1.7–1.8	1.0	1.8	1.7				1.0–1.3	0.8–1.3	

limonene	5.2–9.5			7.6	2.6	2.2	8.9–11.0	1.6	21.2	6.1–7.3	5.19	23.4	12.0–12.7	19.0	14.8	17.7	10.9–12.3		20.0–39.5

1.8-cineole	24.2–25.5	12.7–19.6	12.6–29.8	29.2	24.3	21.8	32.5–37.5	27.2	18.0	24.2–24.6	18.9	11.6	21.6–23.0	26.3	24.6	29.1	21.8–16.6	10.5–18.2	

α-terpinolene															4.8				

β-linalool	2.9–11.7	7.0–15.8	10.8–18.3		1.3	2.9	1.7–2.3	11.8	10.6	2.3–2.5	2.83	20.2	7.5–7.6	6.8	8.3	5.5	8.8–13.4	16.3–18.6	

*p*-menth-1-enol								7.0											

α-terpineol	2.8–3.6	1.6–2.9	3.9–6.6	4.2	2.5	5.7			3.1					1.1			2.1–2.9	4.3–6.5	2.7–5.2

myrtenol		0.8–3.5																	0.8–2.9

methyl chavicol													1.2–1.4						

geraniol					0.0	1.48			1.1									1.3–1.9	

linalyl acetate	0.7–2.9	2.5–6.0	2.7–7.0					3.4	4.6				1.8–2.0	2.2	3.4	2.5	2.6–2.8	5.5–6.8	

myrtenyl acetate		23.7–39.0	13.5–30.7	35.9			14.8–21.1	4.2					14.0–15.2	2.0			11.4–12.3	10.8–14.5	7.0–37.6

bornyl acetate											2.2								

α-terpinyl acetate	1.1–1.6				0.9	1.2	3.7–4.4	1.4	1.3	1.7–1.9	1.87		2.0–2.1	1.6	1.0			0.8–2.6	

eugenol					0.0	4.1													

neryl acetate		0.4–3.1	≤1.6					2.9										0.8–1.1	

geranyl acetate		0.3–1.8	1.4–5.3	1.7	2.1	3.8	1.8–2.2			1.7–2.1	1.4		2.2–2.9	1.0			0.9–1.3	4.2–5.5	

methyl eugenol	0.8–1.1		0.7–2.3		2.3	5.4			1.6								1.6–1.9	1.3–1.8	1.7–4.3

eugenyl methyl ether				2.3															

β-caryophyllene					0.9	1.9													1.7–2.9

**Table 4:** Antioxidant activity of fresh myrtle leaf essential oils extracted by hydrodistillation (HD-EO) and Solvent-Free-Microwave-Extraction (SFME-EO) and other antioxidant references.

Samples	IC50 (μg/mL)
HD-EO	768 ± 83
SFME-EO	693 ± 92
BHT	8 ± 2
Quercetin	3 ± 1
Gallic acid	1.1 ± 0.8

**Table 5:** Comparison of minimum inhibitory concentrations of hydrodistillation (HD-EO) and Solvent-Free-Microwave-Extraction (SFME-EO) using microdilution method.

Microorganism-tests	minimum inhibitory concentration (MIC) (μL/mL) Bainem Algerian fresh myrtle leaf essential oils (100 mg/mL)	

	HD-EO	SFME-EO
**Gram-positive Bacteria**		
*Bacillus subtilis* (ATCC 6633)	20	10
*Staphylococcus aureus* (CIP 7625)	30	20
*Listeria monocytogenes* (CIP 82110)	30	30

**Gram-negative Bacteria**		
*Klebsiella pneumoniae* (E40)	30	20
*Escherichia coli* (E52)	30	10
*Salmonella enterica* (E32)	30	20
*Enterobacter cloacea* (E13)	30	20
*Pseudomonas aeruginosa* (CIP A22)	30	20
**Yeast**		
*Candida albicans* (IPA 200)	50	50
**Fungi**		
*Aspergillus flavus*	50	20
*Aspergillus ochraceus*	30	10
*Fusarium culmorum*	30	10
